# Defining the Construct of Synthetic Androgen Intoxication: An Application of General Brain Arousal

**DOI:** 10.3389/fpsyg.2018.00390

**Published:** 2018-03-29

**Authors:** Tom Hildebrandt, Ashley Heywood, Daniel Wesley, Kurt Schulz

**Affiliations:** Eating and Weight Disorders Program, Department of Psychiatry, Icahn School of Medicine at Mount Sinai, New York, NY, United States

**Keywords:** anabolic steroids, brain arousal, drug dependence, inhibitory control, drug intoxication, impulsivity, body image disturbances, exercise

## Abstract

Synthetic androgens (i. e., anabolic-androgenic steroids) are the primary component to the majority of problematic appearance and performance enhancing drug (APED) use. Despite evidence that these substances are associated with increased risk for aggression, violence, body image disturbances, and polypharmacy and can develop a pattern of chronic use consistent with drug dependence, there are no formal definitions of androgen intoxication. Consequently, the purpose of this paper is to establish a testable theory of androgen intoxication. We present evidence and theorize that synthetic androgen intoxication can be defined by a pattern of poor self-regulation characterized by increased propensity for a range of behaviors (e.g., aggression, sex, drug seeking, exercise, etc.) via androgen mediated effects on general brain arousal. This theory posits that androgens reduce threshold for emotional reactivity, motor response, and alertness to sensory stimuli and disrupt inhibitory control over the behaviors associated with synthetic androgen use. These changes result from alteration to basic neurocircuitry that amplifies limbic activation and reduces top-down cortical control. The implications for this definition are to inform APED specific hypotheses about the behavioral and psychological effects of APED use and provide a basis for establishing clinical, legal, and public health guidelines to address the use and misuse of these substances.

## Complexity vs. severity of appearance and performance enhancing drug use

The core phenomenon of appearance and performance enhancing drug (APED) use is the utilization of a range of substances, selected for specific pharmacological effects on muscle, adipose tissue, and various molecular targets that promote endurance or recovery (e.g., red blood cell production), in sequenced patterns (i.e., “cycles”). Large scale surveys and detailed in-person interviews (Hildebrandt et al., 2007, 2011b; Ip et al., 2011; Westerman et al., 2016) suggest that almost all regular APED users take some form of synthetic androgen (i.e., anabolic-androgenic steroids, selective androgen receptor modulators, etc.) chemically designed to target the androgen receptor (AR) or the production of endogenous AR ligands. In addition to synthetic androgens, APED users often take fitness supplements that include stimulants and other substances that offer additional potency to the effects of the core drug regimen. These supplements may be contaminated with synthetic androgens or other potent chemicals (Abbate et al., 2015), and include a wide range of stimulants that have their own abuse potential (Sanjuan et al., 2016). Other notable substances include insulin and growth factors (e.g., human growth hormone, insulin-like growth factor, etc.) as well as thyroid medications (e.g., thyroxine, etc.) and beta-2 agonists (e.g., clenbuterol), but their use is infrequent and even less likely to be used in isolation from the base of synthetic androgens (Hildebrandt et al., 2007). Ancillary drugs are also common and taken to treat side effects of training and APED use, including the sexual side effects, pain related to training, sleep disturbances, and recovery of the hypothalamic pituitary gonadal (HPG) axis (Hildebrandt et al., 2006). We have argued that the degree of polypharmacy is one marker of problematic APED use (Hildebrandt et al., 2011a) and provided some empirical data to support this finding (Hildebrandt et al., 2007). The majority of these substances originate from “black market” sources and users often deal with some uncertainty about the exact chemical nature of the substances they purchase, as well as their quality, despite a fair number of the most popular synthetic androgens being produced pharmaceutically for medical purposes (e.g., veterinary or wasting diseases).

A critical consequence of this polypharmacy for researchers, policy makers, and clinicians is the open debate about how to define the severity of this drug use and the development of tools and criteria for assessing problematic use. Unlike other classic drugs of abuse such as cocaine and alcohol, APED use lacks a well-defined intoxication syndrome. This absence limits both the development of research and clinical tools to understand, treat, and prevent APED use, but it also has public health implications for legislation, forensics, and criminology. Much of the theoretical work done in this domain has relied on existing addiction-focused diagnostic frameworks (Kanayama et al., 2009b). Despite a well-known set of physical, cognitive, and behavioral consequences to APED use (Nieschlag and Vorona, 2015; Piacentino et al., 2015), an acceptable delineation of intoxication and euphoric/rewarding effects remains absent, even in diagnostic and clinical descriptions of the psychiatric complications to APED use. The default approach has been to link primary reinforcement of APED use to the effects of these substances on outward appearance or athletic performance; a process that *also* requires weeks to months of intense physical exercise and diet to manifest itself. In short, these substances are not abused in isolation of a host of exercise and dieting behaviors that complicate defining a drug-specific intoxication effect and limit the theoretical connection to biologically mediated reward properties of the drug. The latter is the hallmark of substances (and behaviors) classified under the umbrella of addiction or substance use disorder (Koob and Volkow, 2016; Kim et al., 2017).

Worldwide, ~6.4% of males and 1.6% of females have used synthetic androgens (Sagoe et al., 2014). Approximately 1/3rd of all active users report dependence on synthetic androgens (Brower et al., 1991; Brower, 2002; Perry et al., 2005; Kanayama et al., 2009a,c; Skarberg et al., 2009; Hildebrandt et al., 2011b), which places synthetic androgens among the most addictive of all psychoactive substances (Wagner and Anthony, 2002, 2007; SAMSAH, 2008). This dependence liability has been documented with self-report questionnaire studies (Brower et al., 1991; Perry et al., 2005) unstandardized interviews (Midgley et al., 1999; Copeland et al., 2000; Kanayama et al., 2009c), and formal diagnostic interviews (Pope and Katz, 1994; Malone et al., 1995). The evidence of this androgen dependence, largely derived from persistent use in the context of suppression of hypothalamic-gonadal-pituitary (HPG) axis (Kanayama et al., 2010; Christou et al., 2017), stands in stark contrast to evidence of an intoxication syndrome. Consequently, much of the addiction model for synthetic androgens remains linked to evidence of physical withdrawal (Kanayama et al., 2015) rather than the euphoric effects common to most drugs of abuse.

By definition, *intoxication* refers to a clinically significant alteration to behavior or psychology that develops in proximity to drug use (American Psychiatric Association, 2013). Although expectancy and contextual effects can moderate the magnitude of change and specificity of behavioral response to a substance (Finnigan et al., 1995; Gunn et al., 2017), intoxication emerges from effects on the reward system. The nature of intoxication varies by substance (see Table 1), but all of these effects are observed within a short time frame (min to hours). The period of intoxication for synthetic androgens is less clear. Due to the slow metabolization and prolonged exposure periods, establishing thresholds, and models for intoxication is more difficult, complicated by the contextual and expectancy effects of synthetic androgen use, and the co-occurring engagement in rewarding activities (e.g., exercise, drug/alcohol use, etc.).

**Table 1:** Summary of intoxication syndromes for substances.

**Substance**	**Physiological/behavioral/psychological**	**Biological mechanisms**	**Duration of exposure**
Opiates	Drowsiness (may progress to coma) Slurred speech Pupillary constriction (If overdose, pupillary dilation and depressed respiratory rate Initial euphoria followed by apathy Dysphoria Psychomotor agitation/retardation Impaired judgment Impairment in attention or memory Hallucinations (rare) Inattention to environment	Opioid mu receptor	Minutes to hours
Alcohol	Slurred speech Incoordination Unsteady gait Nystagmus Stupor or coma Mood lability (emotion dysregulation) Impaired judgment Impairment in attention or memory	GABA receptors	Minute to hours
Cocaine/stimulants	Hyperactivity Excessive friendliness Grandiosity Hypervigilance Heightened alertness Blunted affect if used chronically Social withdrawal after use Auditory hallucinations Paranoid thoughts Euphoria Anxiety Anger Social sensitivity Impaired judgment	Dopamine serotonin norepinephrine	Seconds to minutes
Cannabis	Impaired motor control Sedation/lethargy Inappropriate laughter Social withdrawal Euphoria Anxiety Impaired judgment Difficulty with complex mental tasks Problems with short-term memory Distortion in sensory perception Slowed perception of time dry mouth tachycardia increased appetite conjunctival injection	eCB receptors	Minutes to hours
Synthetic androgens	Aggressive (in response to provocation) Increased sex drive Reduced sleep Polypharmacy/drug seeking Excessive exercise Increased lean muscle mass Decreased inhibitory control	Androgen receptor Opioid (indirect)	Days to months

*Sources: American Psychiatric Association (2013), McCrady and Epstein (1999)*.

### Establishing a testable definition of androgen intoxication

We propose the term “*androgen intoxication*” to describe the core behavioral and psychological phenomena associated with APED use. Formally, we define this construct as:

A pattern of disturbed self-regulation and self-perception that manifests by an increased emotional sensitivity or arousal, behavioral disinhibition (particularly in response to threat), and valuation of primary (e.g., sex, food, exercise, drugs/alcohol, etc.) and secondary (e.g., money, professional achievement, etc.) reinforcers that impair relationships, decision making, or other areas of functioning in the context of regular use of synthetic androgens.

There are several unique elements to this definition that deviate from intoxication syndromes characterized for alcohol or other drugs of abuse. Our definition excludes a state of euphoria as no reliable interoceptive experience exists for acute androgen use (Su et al., 1993; Fingerhood et al., 1997) and the desired effects (i.e., changes in appearance or athletic performance) do not occur from drug use alone. In addition, we intend our definition to include drug-induced disturbances in both self-regulation and self-perception because androgen-based changes are likely to prime an individual's central nervous system for immediate responses to particular stressors or triggers (e.g., threat) and also alter trait-level beliefs about one's own value or abilities. Impairment in one's life may emerge from either (or both) of these changes. Extending from these fundamental disturbances, our definition includes a number of intermediate shifts in emotional, cognitive, and motivation—reward systems that have the potential for functional impairment. Clinically, this definition allows clinicians to conceptualize changes in the likelihood of these behaviors based on the context in which the individual uses these substances. For instance, androgen intoxication may increase the likelihood of a more aggressive response to threat of loss in a competitive sport or altercation from interpersonal threat (e.g., insult from stranger), but can also explain the absence of aggressive behavior when these cues are not present in the individual's environment.

The purpose of establishing a defined “androgen intoxication syndrome” is to promote research, guide clinical evaluation, and inform treatment, policy, or forensic decisions on APED use. For instance, forensic analysis of drug-facilitated sexual assault requires some clear definition of the intoxication effects likely to impair judgment, reduce inhibition, and otherwise alter the likelihood of the assault (Anderson et al., 2017). Despite this parallel, we acknowledge that there is unlikely to be a reliable threshold (e.g., blood alcohol content above 0.8) for establishing intoxication for synthetic androgens. Rather, the shift in emotion, cognition, and behavior is more likely to be observed in specific contexts (e.g., in threatening situation, in proximity to rewards like sex or drugs, or decisions that involve risk assessment).

## General brain arousal and androgen effects on central nervous system

General brain arousal is a theory that builds on the inherent and systematic redundancy characteristic of information (chemical, electrical, etc.) and its communication through cells within the central nervous system. The theory, described in detail by Pfaff (2006), outlines a testable set of hypotheses about how the CNS prepares for the challenges of navigating the environment while satisfying natural drives for survival (e.g., feeding, sex, etc.). He states:

“General Arousal is higher in an animal or human being who is: (S) more alert to sensory stimuli of all sorts, and (M) more motorically active, and (E) more reactive emotionally (p. 5).”

Fundamental to this definition are the mechanisms within the central nervous system that may be vulnerable to exogenous disturbance (e.g., synthetic androgen use) or pre-existing vulnerability (e.g., genetic or stress-adapted changes) to neuronal communication and responsivity. It also allows for prediction of neuronal and behavioral response based on environmental cues or contexts and the prioritization or devaluation of specific types of information to satisfy the drive or goal (e.g., devalue information about pain for goal of reward).

### Molecular mechanisms of androgens effects on general arousal

Androgen effects in the central nervous system operate through ARs both in cellular membrane and through nuclear receptors, consequently offering both acute and long-term changes to cellular function and the propensity for a range of behaviors. Endogenous androgens originate from both peripheral and central conversion of cholesterol to testosterone (and other neuroactive steroids including dihydrotestosterone, estrogens, prostilgands) through a number of enzymatic steps (Reddy, 2010). The AR gene, comprised of eight exons, has a central DNA binding terminal and N-terminal transactivation domain, and C-terminal ligand binding domain (Matsumoto et al., 2005). Androgens engage ARs then act on neurons to affect acute neuronal firing or through nuclear receptors to alter long-term changes in cellular function, ultimately contributing to performance of a range of neurotransmitter, peptide, inflammatory, and neurotrophic systems. Additionally, androgens metabolize into estrogens which affect arousal through estrogen receptor (ERα/β) mediated effects along the same arousal pathway, providing androgens with a proximal regulator of estrogen mediated effects in the CNS (Bondesson et al., 2015). Consequently, androgens influence arousal directly through AR mediated effects and indirectly through regulation of acute and tonic levels of estrogen availability in the brain (Charlier et al., 2010).

Genetic polymorphisms of ARs/ERs have reliable relationships to behavioral phenotypes observed among humans that include sexual behavior/arousal, affective liability, impulsivity, aggression, hyperactivity, anxiety, and compulsivity (Sundermann et al., 2010; Maney, 2017). Knockout models of ARs/ERs support the essential role of androgens in arousal mediated phenotypes (Hill and Boon, 2009; Kerkhofs et al., 2009), although much of this phenotypic research in the CNS has focused on sexual arousal/behavior (Santi et al., 2017). In addition to polymorphisms, the complex range of co-activator and co-regulators influence transcriptional activity of these nuclear receptors (Li and Al-Azzawi, 2009), the effects of which in the brain have only recently come under investigation (Charlier et al., 2005, 2015; Qiu et al., 2016).

A range of neurotransmitter and neuroendocrine alterations to cellular function occur in the context of synthetic androgen use. Among the most studied are the effects of androgens on GABAergic forebrain neurons responsible for inhibitory control. These changes involve acute, short term disruption of GABA(A) receptor activation and downregulation in receptor expression over longer periods of exposure (Henderson et al., 2006). In addition to these neurotransmitter effects, vasopressin is known to be a key modulator of androgen effects by directing androgen action in the hypothalamus to coordinate outward aggression or threat avoidance/anxiety (Morrison et al., 2016a). Synthetic androgens affect the firing of these vasopressin neurons via indirect effects on serotonergic and dopaminergic pathways within this circuit (Morrison et al., 2016b). Prefrontal—amygdala signaling neurons are also affected via suppression of serotonergic tone (Ambar and Chiavegatto, 2009), suggesting a primary impact on prefrontal—limbic control of behavior in response to salient emotional or rewarding stimuli.

In rodents, self-administration of androgens appears to be mediated centrally (Wood, 2004). In intracerebroventricular (icv) self-administration models, testosterone is self-administered (Wood et al., 2004) and yields increased cFos in medial amygdala, medial preoptic area, and the ventral tagemental area (VTA; Dimeo and Wood, 2006). This synthetic androgen self-administration is blocked by naltrexone as are the physiological markers of androgen intoxication in rodents (depression of respiration, locomotion, and body temperature) (Peters and Wood, 2005). Other evidence of central opioid effects originate from PET imaging of kappa opioid receptor (KOR) in rats administered nandrolone decanoate, which confirm significant up-regulation of the KOR in the caudate/putamen and down-regulation in hypothalamic nuclei, amygdala, NAc, and globus pallidus (Magnusson et al., 2009). These alterations may in part be related to the specific effects of nandrolone on metabolism of dynorphin into hexapeptide Leu-enk-Arg^6^, which has selectivity for μ and δ opioid receptors. Nandrolone pre-treatment appears to have a region specific impact on this metabolism by an unknown mechanism yielding increases in Leu-enk-Arg^6^ in the NAc, but decreases in the caudate/putamen, hypothalamus, and PAG. The sum effects of these changes are not entirely understood. However, they would appear to favor reduced effects of dynorphin-KOR system in the NAc, but stronger effects in the caudate/putamen. Dynorphin and β-endorphin levels have been found to be increased in cerebrospinal fluid in response to methyltestosterone (Daly et al., 2001, 2003) in healthy controls, supporting opioid mediated reward effects of androgens.

The effects of synthetic androgens on the DA system are less clear. Chronic administration of nandrolone decanoate, a potent synthetic androgen, yields decreased D_1_ and increased D_2_ mRNA as well as equivalent changes in the density of these neurons in the caudate/putamen, VTA, and NAc (Kindlundh et al., 2001, 2003). PET imaging in rodents indicates that DA transporter also appears to be up-regulated in these regions (Kindlundh et al., 2002, 2004) and is associated with decreased DA metabolism as indicated by lower levels of DA metabolites even after amphetamine challenge (Birgner et al., 2007) in pre-treatment models of nandrolone. Studies of the effects of nandrolone on DA release to cocaine indicate that DA release in the NAc is attenuated by nandrolone pre-treatment (Kurling-Kailanto et al., 2010). However, icv administration of testosterone yields no measurable change in DA release in NAc (Triemstra et al., 2008), which suggests that synthetic androgens are not directly altering DA tone, but alter the responsivity of the DA system to other reinforcers. Consequently, the effects of androgens on DA responsivity may involve potent effects in serotonin and GABA neurons of the hypothalamic nuclei and potentially upstream effects on forebrain and cortical regions involved in processing and responding to natural and secondary reinforcers (Bitran et al., 1993; Henderson et al., 2006).

Non-genomic actions of androgens and estrogens are also well-documented (Kawata et al., 2008). These effects have only recently become a target for investigation in the brain-behavior effects, but some emerging data implicate non-genomic action in the hippocampus on stress responsivity and anxiety (Romeo et al., 2004; Filova et al., 2015). This mechanism is likely to regulate sex differences in development of the stress-response system (Zup et al., 2014), with androgens reducing hippocampal reaction to external stressors via alterations to hippocampal cell survival. Although research in this area continues to develop, the implications of this mechanism are for immediate effects of androgens on the hypothalamic-pituitary-adrenal (HPA) axis and the behaviors primarily or indirectly affected (stress-induced behavioral responses).

In summary, synthetic androgens appear to alter prefrontal inhibitory control circuits via effects on serotonergic and GABAergic neurotransmitter systems, alter the sensitivity to reward via changes to opioid and dopaminergic transmitter systems, and alter long-term responsivity in HPA axis via both genomic and nongenomic actions. Future research on whether these systems can be “rescued” pharmacologically or what individual differences in experience (e.g., exercise, stress, etc.) may increase risk or resilience to these drugs will help improve clinical efforts to reduce risks related to intoxicating among these individuals.

### Neurocircuitry of androgen effects on general arousal

There is no single identified neurocircuit for general brain arousal, although the influence of androgens (and indirectly estrogens) on brainstem and subcortical neurons (Hamson et al., 2004) is likely a significant contributor to a number of networks important to arousal (Lakke, 1997). In addition, to the effects of androgens on these primary neurons, the presence of ARs/ERs are well-documented in the hypothalamic and amygdala circuits involved in motivated action and neuronal responsivity to environmental cues such as threat, food, or sex (Simerly et al., 1990, 1997; Guerriero, 2009). The effects of ARs/ERs are widespread, ranging from inhibition/activation of neuronal transmission to structural differences that are often conceptualized as masculinizing or defeminizing (Tsai et al., 2015). The hypothalamic and amygdala based circuits have the most direct relevance to models of steroid intoxication as we conceptualize the androgen effects to catalyze greater pre-limbic/limbic influence over behavioral responses. In this conceptualization, the primary intoxication effects are likely to result from decreases in “top-down” cortical control related to parallel activation of limbic arousal that overwhelms existing cognitive inhibitory systems.

The executive and emotional processing of inhibition to emotionally salient or drive specific (i.e., food, sex, threat) environmental cues is well-defined in the literature. The specific neurocircuitry of inhibition involves a “top down” network that integrates a stop-signal from dorsolateral prefrontal cortex (dlPFC) to the striatum and parallel sensory pathway that cycles directly through inferior frontal gyrus (IFG) to the subthalamic nucleus (Wiecki and Frank, 2013). The dlPFC pathway involves stored information about task relevance (e.g., procedure for approaching cue), receiving information from pars orbitalis and visuomotor input from parietal association corticies to exert goal-directed control by biasing sensimotor regions (Miller and Cohen, 2001; Egner and Hirsch, 2005) and releasing frontal operculum (FOp) from inhibitory control (Stevens et al., 2007). The amygdala serves as a primary node in the sensory pathway, engaging bi-directional connections with insula, striatum, and projecting this information to prefrontal regions to affect the emotional biasing of behavioral responses and receiving modulatory executive information from this network. The results of these androgen mediated effects are a higher propensity to act when presented with either (a) salient cue in the environment (e.g., threat, reward, etc.) or (b) internal sensory cue (e.g., hunger, thirst, sexual arousal, strong emotion, etc.).

This neurocircuitry model implies that synthetic androgen use leads to increased excitability of the amygdala, striatum, and insula network leading to increased connectivity with IFG. Other effects may occur within the medial preoptic area (mPOA)/anterior hypothalamus and bed nucleus of the stria terminalis (BNST), which further influence cue valence (e.g., threat perception, reward) and coordinate hypothalamic input to sexual behavior (Jin and Yang, 2014). Simultaneously, androgens are believed to weaken serotonergic regulation of inhibitory control via (a) hypothalamic related projections to the prefrontal, orbital, and inferior frontal cortex (Bethea et al., 2014, 2015), (b) dopamine production via dopamine metabolism in the VTA (Kuhn et al., 2010), and changes to morphology of dendritic spines in DA neurons within the nucleus accumbens shell (shNAc; Wallin-Miller et al., 2016). The latter effects increase the sensitivity of the motivation reward system, in part by reducing dopaminergic tone, and would affect decision making choices related to reward.

Networks of the extended amygdala appear to be heavily influenced by androgens and stimulation of these networks via synthetic androgens are likely to include both alterations in top-down regulation of the limbic system and increased influence of these subcortical nodes on larger cognitive networks involved in self-regulation and behavioral inhibition. Leverage of neuroimaging methods in humans in the natural course of their APED use may help develop a more specific neurocircuitry of steroid intoxication.

### Cognitive effects of androgens via general arousal

#### Inhibitory control and response inhibition

There are very few data available on active synthetic androgen users examining cognitive measures of inhibitory control. Visuospatial learning and memory appear to be lower in long-term synthetic androgen users and positively related to lifetime exposure to synthetic androgens (Kanayama et al., 2013), but there is also evidence of larger amygdala volumes and weakened connectivity between amygdala and NAc, IFG, dlPFC, and thalamus (Kaufman et al., 2015). The only large scale imaging study of chronic synthetic androgen users suggest greater reductions in cortical thickness, total gray matter, and many specific subregions, but failed to replicate the volumetric differences in amygdala found in a previous study (Bjørnebekk et al., 2017). In small study of synthetic androgen users, our lab found that adolescent onset users showed greater impairment in inhibitory control, particularly when they were on-cycle relative to adult onset users (Hildebrandt et al., 2014). These data are consistent with rodent studies of synthetic androgen exposure that demonstrate that adolescent steroid exposure yields persistent elevations in aggression, but that adult exposure only alters aggressive response under the direct influence of these androgens (Cunningham et al., 2013). Consequently, the influence of these substances may depend on normal developmental changes in inhibitory control networks of the brain.

### Behavioral effects of androgens via general arousal

#### Aggression, violence, and criminality

The psychological effects of synthetic androgens are well-documented and can take the form of increased tolerance of pain, valuation of exercise, reward sensitivity, and propensity for aggression (Hildebrandt, 2013) that is the primary contributor of the two to five-fold increased risks in violence, violent death, suicide, and major crime of synthetic androgen users compared to others illicit substance users (Thiblin et al., 2000; Petersson et al., 2006; Klötz et al., 2007). The developmental significance of steroid intoxication is largely unknown, but some observational data suggest that adolescent onset steroid use is associated with greater risk for criminality and violence (Irving et al., 2002; vandenberg et al., 2007), that this pattern persists after drug initiation (Skårberg et al., 2010), and may not act proximally, but rather conveys an increased relative risk compared to other drug users and incarcerated criminals (Lundholm et al., 2010). Emerging data also suggest that vulnerable individuals, including those who have already displayed aggressive behavior, in adolescence are likely to demonstrate antisocial and violent behavior (Hallgren et al., 2015).

#### Comorbid substance abuse and polypharmacy

There are significant correlations in large survey studies between frequency of substance use/misuse and synthetic androgen exposure (Lundholm et al., 2015; Sagoe et al., 2015). Studies of gym attendees who use synthetic androgens support these data, suggesting a greater risk for using other drugs of abuse including alcohol, marijuana, cocaine, and opioid/pain killing substances (Ip et al., 2011; Schwingel et al., 2014; Molero et al., 2017; Struik et al., 2017). As noted above, there is some experimental evidence to support the sensitivity to amphetamine, cocaine, and opiate drugs when taking a synthetic androgen. More recent data indicate that chronic androgen administration can suppress endocannabinoid tone in the CNS, altering the potential rewarding properties of a range of substances (Struik et al., 2017). Structural imaging studies also suggest that polypharmacy may exacerbate reductions in cortical thickness, gray matter volume, and integrity of white mater tracts in the inferior frontal regions (Bjørnebekk et al., 2017; Seitz et al., 2017). Taken together, these data largely suggest that androgen misuse in humans and experimental models with animals alter the sensitivity to these rewards and chronic use may result in structural brain changes that affect reward processing and inhibition.

#### Body image, dieting, and exercise

The relationship between disturbances in body image and synthetic androgen use are well-documented and considered to a primary contributor to the dependence syndrome (Piacentino et al., 2015). This contribution is perhaps best defined by an over *investment* in outward appearance as opposed to negative *evaluation* of appearance. Although both occur among synthetic androgen users, the separation allows for the observation that many who use these substances are satisfied with their appearance (Hildebrandt et al., 2010). Rather, those who have a heavy investment in appearance are more likely to also control their dieting practices and experience functional impairment related to their substance use (Hildebrandt et al., 2010; Murray et al., 2016). This constellation of attitudes also supports our definition of intoxication in that elevated importance of appearance (i.e., greater valuation of one's body) would contribute to greater sensitivity and decreased inhibitory control over behavioral response to body threats and body rewards. This imbalance in valuation and disinhibition of body related control could occur via increased general arousal changes that originate from molecular and neurocircuitry changes in prefrontal—limbic regions that govern response to threat and reward.

In addition to sensitivity to body specific threats and rewards, exercise has a critical role in the intoxication related effects of androgens. To achieve the desired effects of these substances, a significant amount of time and effort must be devoted to exercise. This requires both an increased tolerance for pain of heavy exercise and psychomotor activation to engage in this behavior. We have posited that synthetic androgens achieve this effect by altering the allostatic response of the body to exercise stress (Hildebrandt et al., 2011c). In this model, androgens reduce the cortisol response to exercise, while enhancing the endorphin response. This conceptualization also reduces the issues for intoxication related to proximity of the reward. If androgens increase the responsivity of the CNS to rewards, specifically exercise related rewards through a central mechanism, then the temporally reinforcing effects of androgens are more proximally related to euphoria specific to exercise than drug administration. In a pilot test of this theory, we found that on-drug androgen users were more motivated to earn access to exercise than off-drug androgen users or heavy exercising controls. This motivation was correlated with plasma endorphin levels, suggesting some connection between exercise and reward system that is mediated by synthetic androgens.

## Applying general brain arousal to steroid intoxication

**Figure 1:**
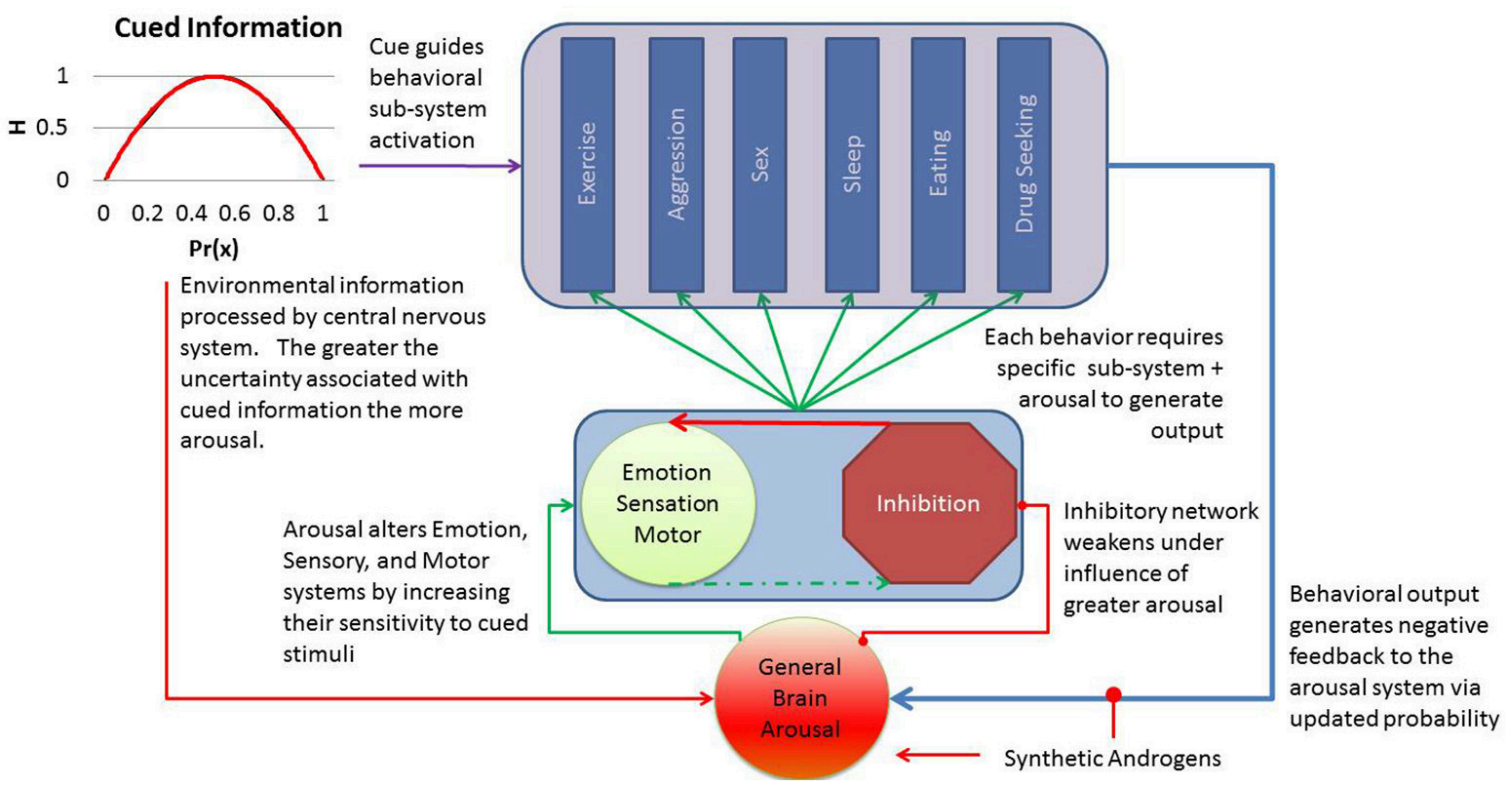
The general arousal model of steroid intoxication positions the central nervous system to receive information from internal or environmental cues. This information is often represented as a function of the probability of cue (x) and the degree of uncertainty or entropy (H). As this information activates the arousal network, primed by synthetic androgens, an individual's threshold for emotional reactivity, sensory stimulation, and action decrease and allow the cue to generate a range of behaviors more quickly. Although healthy arousal should diminish after expression of the behavior, the artificial amplification of arousal reduces this natural negative feedback mechanism and results in greater intensity, magnitude, and frequency of the output behavior. Individual differences in arousal or inhibitory control should explain resilience or vulnerability to these androgen effects.

Figure 1 summarizes our general model for intoxication, drawing from the theory on general brain arousal to explain the range of behaviors with higher propensity for occurring when cued by some environmental or internal stimulus. We hypothesize that steroid intoxication arises from direct effects on general brain arousal that reduce the threshold for cued information to generate a behavioral response. The behavioral response depends on its unique subsystem for execution, but termination of the behavior offers some negative feedback to a healthy general arousal system. This negative feedback helps to reduce the propensity for other behaviors by altering the input of information. However, the exogenous influence of androgens keeps arousal high and limits the impact of this natural negative feedback. For example, a threat cue would drive brain arousal (not autonomic, this would be a subsystem response) and increase the propensity for a host of behavioral responses. The nature of threat would bias specific a subsystem responses (e.g., toward aggression) to the cue, creating a context specific behavioral response (e.g., aggressive response to threat). The inhibitory network response diminishes as arousal drives up emotion, alertness, and motor activation resulting in release of the subsystem involved in guiding the specific response to action. The arousal system primed by synthetic androgens support greater magnitude, duration, and intensity of the behavioral output from the specific subsystem (e.g., more aggression, less inhibition of response, greater valuation of the cue).

As highlighted in our model, general arousal offers a mechanism by which synthetic androgens can drive up the propensity for a host of impulsive behaviors by limiting the inhibitory control over cued responses to aggression, sexual behavior, reduced sleep, eating, increased locomotion/exercise, or drug seeking. This model explains why these behaviors vary by individual as both environmental exposure and individual differences in each behavioral subsystem may alter propensity of a specific behavior. In addition, it yields several testable hypotheses about synthetic androgen use that could help us develop predictive models for the effects of steroid intoxication.

1. The *covariance* of these behaviors should be greater under the influence of synthetic androgens than during pre or post-cycle androgen states.
2. The *propensity* for these behaviors will increase as a function of increases in emotional responsivity, sensory alertness, and motor activity.
3. The inhibitory response network will be less active in response to salient cues (e.g., threat, reward, etc.) in a state of steroid intoxication.

The additional complexity of human intoxication syndromes can be added to this brain arousal model by accounting for amplifying/suppressing effects of the substances on arousal and inhibitory control. The majority of substances used in polypharmic versions of APED use would amplify this general arousal effect (e.g., stimulants), with notable exceptions (e.g., opioid medications for pain). The persistence of this arousal, maintained by supraphysiological doses of androgens, also explains some of the disturbances in sleep and mood that have been observed and described as hypomania or mania in the literature (Pope and Katz, 1994; Pope et al., 2000).

## Conclusions and future directions

Although the translational science of androgen misuse has made great strides (Wood and Peters, 2008; Oberlander and Henderson, 2012; Wood et al., 2013) and the molecular and neuroendocrine effects of androgens are increasingly well-understood in animals (Vicencio et al., 2011; Grosse et al., 2012; Oberlander et al., 2012), application of these pre-clinical data to humans has lagged well-behind other drugs of abuse. The absence of “real world” data on developmental and acute effects of synthetic androgens is often used by the APED using community to justify increasingly dangerous patterns of drug use (Monaghan, 2002). Furthermore, the absence of these data has contributed to the growing divide between health care professionals and APED users (Pope et al., 2004), who experience significant stigma from the health care community. The creation of a testable intoxication syndrome, based on the changes in brain arousal that affect the propensity for a wide range of behaviors based on decreased threshold for sensory, motor, and emotional activation to relevant cues, should help delineate risks attributable to the substances and guide policy makers and public health efforts.

In addition to adoption and testing of meaningful intoxication syndrome, identification of those with pre-existing vulnerability in the brain arousal system may help the field move toward individualized risk profiles. A clear example of this type of risk analysis emerges from the trait like changes that emerge from adolescent exposure in rodents and preliminary data from our lab that suggest changes in function of arousal—inhibitory network may persist past any acute exposure period.

## Author contributions

TH and KS conceptualized the manuscript and TH developed a first draft. AH and DW provided feedback and contributed content for Table 1. All authors agreed to final version of the manuscript.

### Conflict of interest statement

The authors declare that the research was conducted in the absence of any commercial or financial relationships that could be construed as a potential conflict of interest.
